# Characterization of oral biomarkers during early healing at augmented dental implant sites

**DOI:** 10.1111/jre.13328

**Published:** 2024-08-01

**Authors:** Lorenzo Tavelli, Shayan Barootchi, Maria Vera Rodriguez, Jim Sugai, David T. Wu, Ning Yu, William V. Giannobile

**Affiliations:** ^1^ Division of Periodontology, Department of Oral Medicine, Infection, and Immunity Harvard School of Dental Medicine Boston Massachusetts USA; ^2^ Center for Clinical Research and Evidence Synthesis in Oral Tissue Regeneration (CRITERION) Boston Massachusetts USA; ^3^ School of Dentistry Universidad Catolica de Santiago de Guayaquil (UCSG) Guayaquil Ecuador; ^4^ Department of Periodontics & Oral Medicine University of Michigan School of Dentistry Ann Arbor Michigan USA; ^5^ Postgraduate Periodontics, Division of Periodontics Columbia University College of Dental Medicine New York City New York USA; ^6^ Harvard John A. Paulson School of Engineering and Applied Sciences Harvard University Cambridge Massachusetts USA; ^7^ Wyss Institute for Biologically Inspired Engineering Harvard University Boston Massachusetts USA; ^8^ ADA Forsyth Institute Cambridge Massachusetts USA

**Keywords:** blood circulation, blood flow velocity, crevicular fluid, dental implants, graft, ultrasonography, wound healing

## Abstract

**Aim:**

The aim of this study is to assess early wound healing expression of local angiogenic biomarkers following connective tissue graft (CTG) at dental implant sites.

**Methods:**

Twenty‐eight subjects with single dental implants exhibiting a soft tissue dehiscence were included and randomly treated with CTG, either with coronally advanced flap (CAF) or with tunnel technique (TUN). Peri‐implant crevicular fluid (PICF) was collected at the midfacial and midlingual aspect of the implant sites at baseline and at 3, 7, 14, 30, and 90 days after the surgical intervention. The expression of angiogenin (ANG), fibroblast growth factor‐2 (FGF‐2), platelet‐derived growth factor (PDGF), tissue inhibitor of metalloproteinases‐2 (TIMP‐2), and vascular endothelial growth factor (VEGF) was investigated over a period of 3 months. Patient‐reported outcomes, clinical measurements, and ultrasonography scans at multiple time points were also evaluated.

**Results:**

The longitudinal regression revealed a significant difference in the expression of VEGF and TIMP‐2 between CAF‐ and TUN‐treated sites over 3 months (*p* = .033 and *p* = .004, respectively), whereas no significant differences were observed for ANG, FGF‐2 and PDGF between the two groups. At 7 days, a direct correlation was observed between ANG levels and ultrasonographic color velocity in the CAF group (*p* < .001) and between ANG levels and ultrasonographic color power in the TUN group (*p* = .028). VEGF levels and ultrasonographic mean perfused area of the CTG were significantly correlated at the 7‐day time point (*p* < .001 for both CAF and TUN). The expression of VEGF at 7 days was directly associated with mucosal thickness gain at 1 year (*p* < .001 for both groups). Early TIMP‐2 expression showed an inverse correlation with time to recovery (*p* = .002). TIMP‐2 levels at 3 months exhibited inverse correlations with mean dehiscence coverage (*p* = .004) and the rate of complete dehiscence coverage (*p* = .012).

**Conclusion:**

PICF biomarkers can be used to monitor early wound healing events following soft tissue grafting at implant sites. VEGF and TIMP‐2 showed correlations with the 1‐year clinical and volumetric outcomes, as well as with post‐operative patient‐reported outcomes and Doppler Ultrasonographic tissue perfusion‐related parameters.

## Graphical abstract

Dental implants treated with a connective tissue graft, either with coronally advanced flap or with tunnel technique, showed a different expression of VEGF and TIMP‐2. The expression of angiogenic biomarkers at 1 week was correlated to the ultrasonographic tissue perfusion of the graft. Early levels of TIMP‐2 and VEGF predict clinical and patient‐reported outcomes.

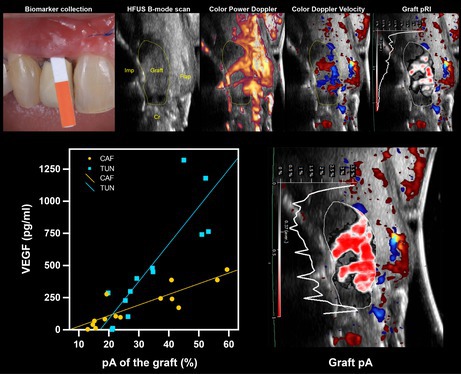

**Clinical relevance**

**Background**

There is the need to explore non‐invasive methods to assess oral wound healing at implant sites. To this aim, the present study used the analysis of PICF biomarker and doppler ultrasonography to monitor early wound healing events following soft tissue grafting around dental implants.

**Added value of this study**

Significant correlations between ultrasonographic tissue perfusion outcomes and the levels of ANG and VEGF were observed at 7 days. The early expression of VEGF was significantly associated with the gain in mucosal thickness at the grafted implant sites at 1 year. Early levels of TIMP‐2 exhibited an inverse correlation with time to recovery.

**Clinical implications**

PICF biomarkers assessment is non‐invasive tools that may be used to monitor the healing following CTG at implant sites. The different expression of VEGF and TIMP‐2 at CAF‐ and TUN‐treated sites further highlights the different wound healing dynamics of the two approaches that may also explain their final outcomes. Early expression of TIMP‐2 and VEGF can predict certain clinical and patient‐related outcomes at 1 year.

## 1 INTRODUCTION

Soft tissue grafting procedures are commonly performed at dental implant sites to re‐establish an adequate amount of keratinized mucosa, increasing soft tissue thickness, and/or treating implant esthetic complications.^1^ Although most of the available studies focused on the 6‐ and 12‐month clinical outcomes of these interventions,^2^ little is known about early wound healing phenomena following peri‐implant soft tissue augmentation.

Oral wound repair is a complex biological process involving a myriad of cell–cell and cell–matrix interactions orchestrated by signaling molecules and cytokines.^3^, ^4^ The initial phases of wound healing are characterized by a rapid cascade of events regulated by signaling molecules, such as angiogenin (ANG), fibroblast growth factor (FGF), platelet‐derived growth factor (PDGF), and vascular endothelial growth factor (VEGF) among others^5^ that contribute to promoting angiogenesis, and organizing the local vascular network at the healing site.^4^, ^6^

Recent development in imaging technologies have enabled clinicians and researchers to monitor and quantify these wound healing phenomena in‐vivo. Fluorescein angiography, laser doppler flowmetry, laser speckle contrast imaging, and high‐frequency ultrasonography (HFUS) have been utilized to investigate the process of revascularization of autogenous soft tissue grafts.^7^, ^8^, ^9^, ^10^

Analysis of extracellular matrix molecules from the gingival or peri‐implant crevicular fluid (GCF and PICF, respectively) has also gained popularity as a non‐invasiveness tool for assessing markers of inflammation, angiogenesis, tissue degradation, and cell proliferation among others.^11^ The evaluation of biomarkers expression at multiple time points has been used to characterize and discriminate wound healing events occurring following different surgical procedures.^11^, ^12^, ^13^, ^14^, ^15^ Pellegrini et al. described the expression of wound healing biomarkers over time at sites treated with a periodontal regenerative approach open flap debridement.^13^ Few studies have previously described the expression of early wound healing biomarkers following soft tissue grafting procedures in natural dentition.^3^, ^12^, ^16^ Morelli and coworkers demonstrated a significant increase of angiogenic biomarkers, including angiostatin, PDGF‐BB, VEGF, FGF‐2 and interleukin‐8 (IL‐8) at teeth grafted with a living cellular construct compared with sites that received a free gingival graft (FGG).^12^ Skurska et al. investigated the concentration of metalloproteinases‐1 (MMP‐1) and metalloproteinases‐8 (MMP‐8) for characterizing the healing process following root coverage procedures with either connective tissue graft (CTG) or a collagen matrix.^3^ Other wound healing biomarkers that have been explored for characterizing the healing of CTG in natural dentition included ANG, VEGF, and tissue inhibitor of metalloproteinases (TIMPs), among others.^16^, ^17^, ^18^ However, the current evidence on the use of protein biomarkers for depicting the healing of soft tissue grafts is limited to clinical studies performed in natural dentition. In addition, it is a reasonable assumption that protein biomarkers may provide novel insights on the wound healing events occurring at implant sites augmented with different soft tissue grafts and/or different surgical techniques (i.e., coronally advanced flap [CAF] vs. tunnel technique [TUN]).

Therefore, the aim of the present study was to utilize angiogenic biomarkers to assess the healing of CTG at implant sites, when performed with CAF or TUN, and to explore the significance of early biomarker expression on the clinical, volumetric, and patient‐reported outcomes of soft tissue augmentation.

## 2 MATERIALS AND METHODS

### 2.1 Study design

The present study was designed as a secondary analysis of a previously reported double‐blind, parallel‐arm, randomized, controlled clinical trial on the treatment of peri‐implant soft tissue dehiscences (PSTDs) using either (CAF + CTG or TUN + CTG ClinicalTrial.gov # NCT03498911).^19^ The study protocol was approved by the Institutional Review Board of the University of Michigan Medical School (HUM00140205) and was in accordance with the Declaration of Helsinki of 1975, revised in Fortaleza in 2013. All participants were informed and understood the aims and the details of the study before signing a written informed consent document. The CONSORT statement was followed in preparing this manuscript.

### 2.2 Participants

Twenty‐eight subjects presenting with isolated healthy dental implants exhibiting PSTDs^20^ associated with esthetic concerns were enrolled and treated with CTG, in combination with either CAF or TUN. Subjects presenting with PSTDs in a non‐molar site were screened at the Department of Periodontics and Oral Medicine, the University of Michigan School of Dentistry (Ann Arbor, USA) between July 2018 and September 2020. Patients satisfying the following inclusion criteria were recruited: (i) Age ≥18 years, (ii) Full‐mouth plaque score and full‐mouth bleeding score ≤20%, (iii) Dental implants with an isolated Class II PSTD, subclass a or b,^20^ located at a non‐molar site and between two natural teeth, (iv) implants characterized by a condition of peri‐implant health, without signs of clinical inflammation,^21^ and (v) Osseointegrated and functionally loaded dental implants that had been prosthetically loaded for at least 12 months.

Exclusion criteria were (i) History of previous PSTD treatment at the implant site, (ii) Systemic conditions that can impair oral wound healing (e.g., uncontrolled diabetes), (iii) Contraindications for surgery, (iv) Natural dentition with one or more sites having probing depth (PD) ≥5 mm, (v) Class I, III or IV PSTDs or subclass c PSTDs,^20^ (vi) Multiple adjacent implants with PSTDs, (vii) PSTDs with implant‐supported crown margin located ≥3 mm apical than the gingival margin of the homologous contralateral tooth, (viii) Diagnosis of peri‐implantitis,^22^ (ix) Previous surgery at the implant site within the past 6 months, and (x) Smoking more than 10 cigarettes a day.

### 2.3 Study outcomes

This study evaluated the expression of ANG, FGF‐2, PDGF‐BB, TIMP‐2 and VEGF over 3 months following soft tissue grafting procedures with the assessment of possible correlations between biomarker expression and Doppler ultrasonographic tissue perfusion, as well as correlations between early wound healing biomarkers, patient‐reported post‐operative morbidity, and the 1‐year clinical outcomes of PSTD treatment.

### 2.4 Biofluid collection and processing

**FIGURE 1:**
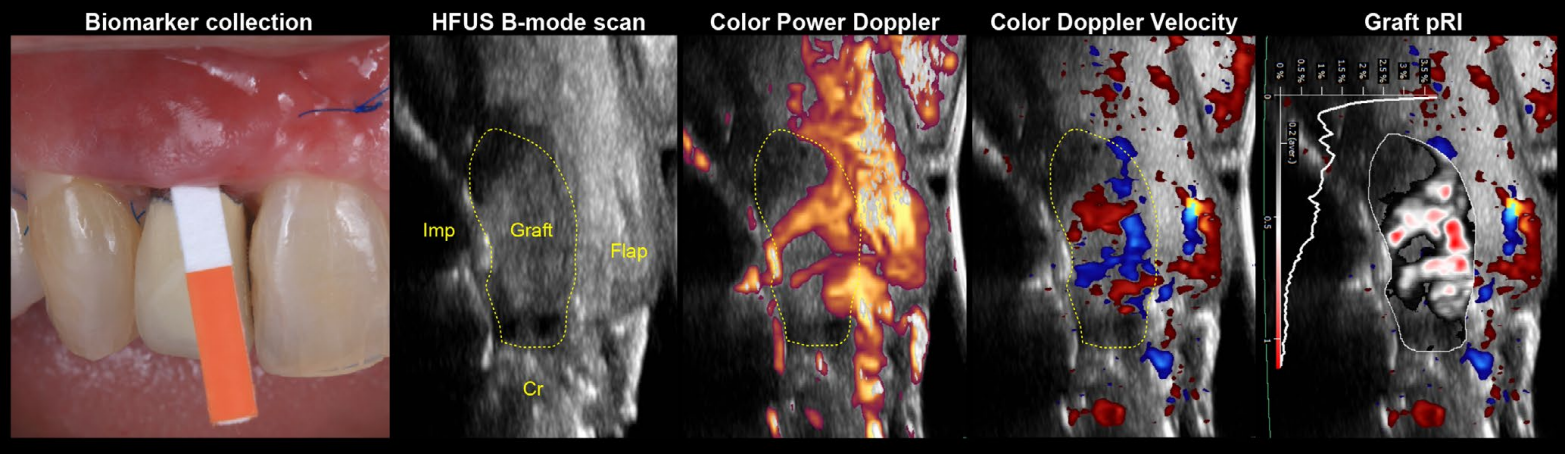
Collection of the peri‐implant crevicular fluid at 1 week and related high frequency ultrasonographic (HFUS) scans recorded at the midfacial aspect. It is possible to appreciate the implant‐supported crown (“Cr”), the implant fixture (“Imp”), the graft, and the flap. Doppler ultrasonographic blood flow evaluation included Color Power Doppler, Color Doppler Velocity, and dynamic tissue perfusion assessment. In particular, the right panel displays the dynamic tissue perfusion evaluation in terms of perfusion relief intensity (pRI) of the connective tissue graft after 1 week. pRI refers to the number of pixel within the region of interest (graft) that relate to the intensity of the respective value. Areas with zero intensity/perfusion within the ROI are shown in black, whereas areas with half of the maximum intensity/perfusion and maximum intensity/perfusion are visualized in white and red, respectively.

PICF was collected from the midfacial and midlingual aspects of the implant site at baseline (the day of the surgery, prior to performing local anesthesia), and at 3 days, 7 days, 14 days, 30 days, and 90 days after the intervention (Figure 1). PICF collection involved the use of methylcellulose paper strips (Periopaper, Oraflow, Smithtown, NY, USA). After air‐drying the area of interest, the paper strip was gently inserted, until slight resistance was felt, into the peri‐implant sulcus at the midfacial aspect, for 30 s.^12^ Each strip was placed in an individually labeled, plastic vial, and stored in a −80°C freezer until the analysis was performed. PICF samples were processed all at once after the conclusion of the clinical portion of the study. A 20‐μL extraction solution (10 mg/mL aprotinin, 1 mM phenylmethylsulfonyl fluoride, and 0.1% human serum albumin in phosphate‐buffered saline) was pipetted onto the cellulose portion of each PICF strip, that was secured at the top of a polystyrene culture tube (12 × 75 mm) using a cap to keep the strip in place. Then, the tubes were centrifuged at 2000 rpm at 4°C for 5 min. Each strip was washed and centrifuged five times to yield a total elution volume of 100 μL. Samples were added to custom human antibody arrays then scanned and analyzed according to the manufacturer's protocol (RayBiotech Life, Inc., Norcross, GA, USA).^11^, ^12^

### 2.5 Clinical, ultrasonographic, and patient‐reported outcomes

Clinical measurements included the apico‐coronal depth of the PSTD, mean PSTD coverage, complete PSTD coverage, and the gain in keratinized mucosa width (KM) from baseline to 12 months (Appendix S1). Ultrasound scans were taken at baseline, 1 week, 1 month, 6 months and 12 months as previously described.^19^, ^23^ Mucosal thickness (MT) gain from baseline to 12 months was assessed on brightness mode (B‐mode) scans (Appendix S1). Tissue perfusion was assessed and recorded as 6 s cine loops of Color Doppler Velocity (CDV) and Power Doppler Imaging (PDI) modalities capturing at least 5 cardiac cycles. CDV mode allows to detect mean velocity of blood flow within vessel through color coding, based on the scattered signal produced by moving erythrocytes that results in a change in frequency of the reflected sounds waves that are received by the ultrasound transducer. This imaging modality provides information on blood flow direction and relative velocity, whereas PDI displays in a single‐hue red color the power/amplitude of the blood flow. CDV and PDI were computed as previously described.^10^, ^24^

In addition, the early dynamic tissue perfusion measurements (DTPMs) were computed on the 1‐week and 1‐month ultrasound scans and reported as mean perfusion relief intensity (pRI), mean perfused area (pA), and mean flow intensity (FI) by defining the graft and the flap as regions of interest (ROIs) after importing the DICOM files in a software package (PixelFlux, Chameleon‐Software, Germany)^23^, ^25^, ^26^ (Appendix S1) (Figure 1).

Patient‐reported outcome measures (PROMs) including post‐operative morbidities, captured during the first 2 weeks after surgery using a 0–100 visual analogue scale (VAS) and the time to recovery, defined as the number of days required to reach a VAS <10,^19^ were also considered.

### 2.6 Statistical analysis

Mean values and standard deviations (SD) were calculated for describing continuous variables. Longitudinal regression models using generalized estimation equations (GEE) were conducted to assess changes of the expression of biomarkers over time according to the surgical technique (CAF vs. TUN). Wald's Chi‐Squared test was used to conclude about main effects and interactions. Pairwise comparisons were adjusted by Bonferroni's correction. Shapiro–Wilk's tests were conducted to assess whether ultrasonographic tissue perfusion‐related parameters, mean PSTD coverage, KM, MT and VAS changes fitted to normal distributions, concluding some significant deviations. Non‐parametric Spearman's correlation and Mann–Whitney's test were used to assess relationships and homogeneity between groups. Regression models using GEE were performed to evaluate relationships between biomarker expressions and clinical outcomes. Significance level used in the analysis was 5% (α = 0.05). All analyses were performed by an independent biostatistician, who had not taken part in the clinical portion of the study.

## 3 RESULTS

### 3.1 Study participants and characteristics at baseline

Twenty‐eight subjects (mean age 47.0 ± 12.1 years, 16 females and 12 males), each contributing with a single dental implant, were allocated and treated with CTG, in combination with either CAF or TUN. All subjects completed the appointment visits. Characteristics of the included subjects and implant sites are reported in Table 1.

**TABLE 1:** Participants and site characteristics at baseline.

Parameter	CAF (*N* = 14)	TUN (*N* = 14)
Age (mean ± SD) (years)	46.9 ± 9.8	47.1 ± 13.6
Females (*N*)/(%)	8/57.1	8/57.1
Smokers (*N*)	1^a^	1^a^
PSTD subclasses	7 PSTD subclass a, 7 PSTD subclass b	6 PSTD subclass a, 8 PSTD subclass b
PSTD characterized by exposure of the abutment only (*N*)	10	11
PSTD characterized by exposure of the abutment and implant fixture (*N*)	4	3
Bone level/tissue level implants (*N*)	14/0	14/0
Maxillary/mandibular sites (*N*)	8/6	10/4
Central incisor/lateral incisor/canine, premolar sites (*N*)	4/3/0/7	3/2/1/8
PSTD depth (mean ± SD) (mm)	2.46 ± 0.87	2.36 ± 0.46
KMW (mean ± SD) (mm)	1.96 ± 1.35	1.79 ± 0.99
MT (mean ± SD) (mm)	1.28 ± 0.29	1.56 ± 0.47

Abbreviations: CAF, coronally advanced flap; KMW, keratinized mucosa width; MT, mucosal thickness; PSTD, peri‐implant soft tissue dehiscence; TUN, tunnel technique.

^a^ Both patients have been considered smokers due to their smoking history. However, they stated that they had quit smoking a few months prior to initiating the study and they have not resumed smoking since.

### 3.2 Expression of ANG, FGF‐2, PDGF‐BB, TIMP‐2, and VEGF

**FIGURE 2:**
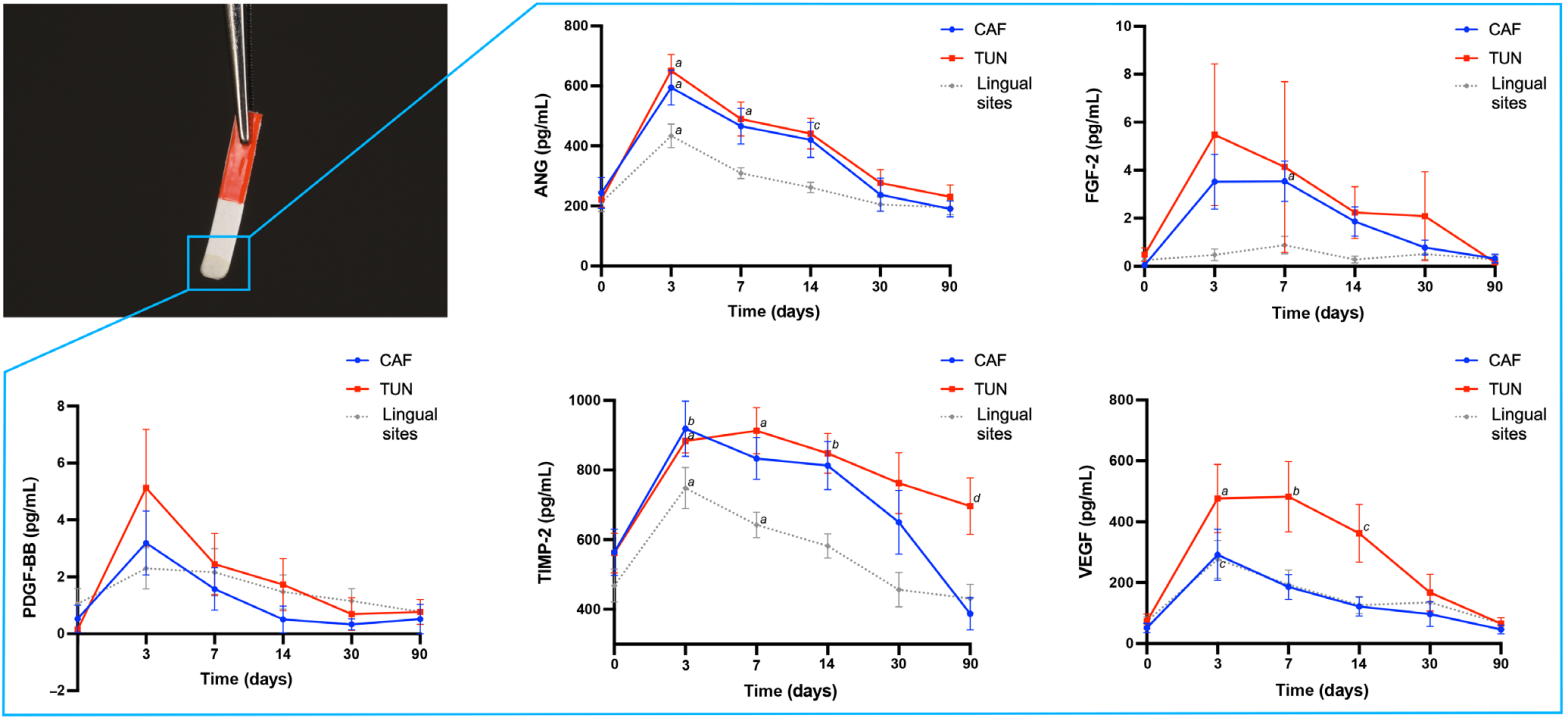
Graphs depicting the variations of ANG, FGF‐2, PDGF‐BB, TIMP‐2, and VEGF at the buccal aspect of implant sites augmented with CTG (either with CAF or TUN), and at their lingual aspect. Mean and standard error reported for each panel. Legend. “a” denotes a statistically significant difference (*p* < .001) between the buccal aspect (either CAF or TUN) and the lingual aspect. “b” and “c” denote a statistically significant difference (*p* < .01 and *p* < .05, respectively) between the buccal aspect (either CAF or TUN) and the lingual aspect. “d” indicates a statistically significant difference (*p* < .05) between CAF and TUN.

Figure 2 and Table S1 of the Appendix S1 depict the expression of the investigated biomarkers from baseline to the 3‐month post‐operative visit. No significant differences were found between CAF and TUN in terms of ANG expression over time (*p* = .701). The longitudinal analysis revealed a significantly different expression of ANG at CAF‐treated sites compared with lingual sites (estimated coefficient of −1.39 (95% CI [−2.45, −0.33]), *p* = .01), whereas a marginally significant difference was observed between TUN‐treated sites and lingual sites (estimated coefficient of −1.15 (95% CI [−2.36, 0.06]), *p* = .063) (Table S2). No differences were found between CAF and TUN from the longitudinal regression analysis (*p* = .547), whereas the CAF group exhibited a different FGF‐2 expression pattern over time compared with lingual sites (estimated coefficient of −0.02 (95% CI [−0.03, 0.01]), *p* = .001) (Table S3). No significant differences were observed among CAF, TUN, and lingual sites in terms of PDGF‐BB expression (Table S4). The CAF group exhibited a significantly faster reduction of TIMP‐2 levels over time compared with the TUN group (estimated coefficient of −3.51 (95% CI [−5.70, −1.32]), *p* = .002). Both groups displayed significant different patterns of TIMP‐2 expression compared with the lingual sites. However, when time was taken into consideration, only the CAF group showed a significantly different expression of TIMP‐2 compared with lingual sites (estimated coefficient of −2.16 (95% CI [−3.61, −0.70]), *p* = .004) (Table S5). VEGF expression over time in the TUN group was found to be significantly different from the ones observed in the CAF group (estimated coefficient of 1.73 (95% CI [0.14, 3.31]), *p* = .033) and at the lingual aspect (estimated coefficient of −1.90 (95% CI [−3.54, −0.26]), *p* = .022) (Table S6).

### 3.3 Biomarker levels and correlation with the 1‐year clinical and patient‐reported outcomes

Regression models revealed statistically significant inverse correlations between TIMP‐2 levels at 3 months and mean PSTD coverage (estimated coefficient of −0.074 (95% CI [−0.122, −0.027]), *p* = .004) and between TIMP‐2 levels at 3 months and the rate of complete PSTD coverage (OR 0.991 (95% CI [0.984, 0.998]), *p* = .012). High values of TIMP‐2 at 3 months were associated with a lower likelihood of achieving complete PSTD coverage. Regression analysis did not show significant correlations between biomarker levels and KM gain at 1 year. The expression of VEGF at 7 days in the two groups was directly associated with MT gain at 1 year (estimated coefficient of 0.001 (95% CI [0.000, 0.001]), *p* = .014). TIMP‐2 expression at 7 days was found to be inversely correlated with time to recovery (estimated coefficient of −0.009 (95% CI [−0.015, −0.004]), *p* = .002).

### 3.4 Biomarker expression and early ultrasonographic graft perfusion

At 7 days, a direct correlation was observed between ANG levels and CDV in the CAF group (*p* < .001). At the same time point, ANG levels were found to be directly correlated with PDI in the TUN group (*p* = .028).

**FIGURE 3:**
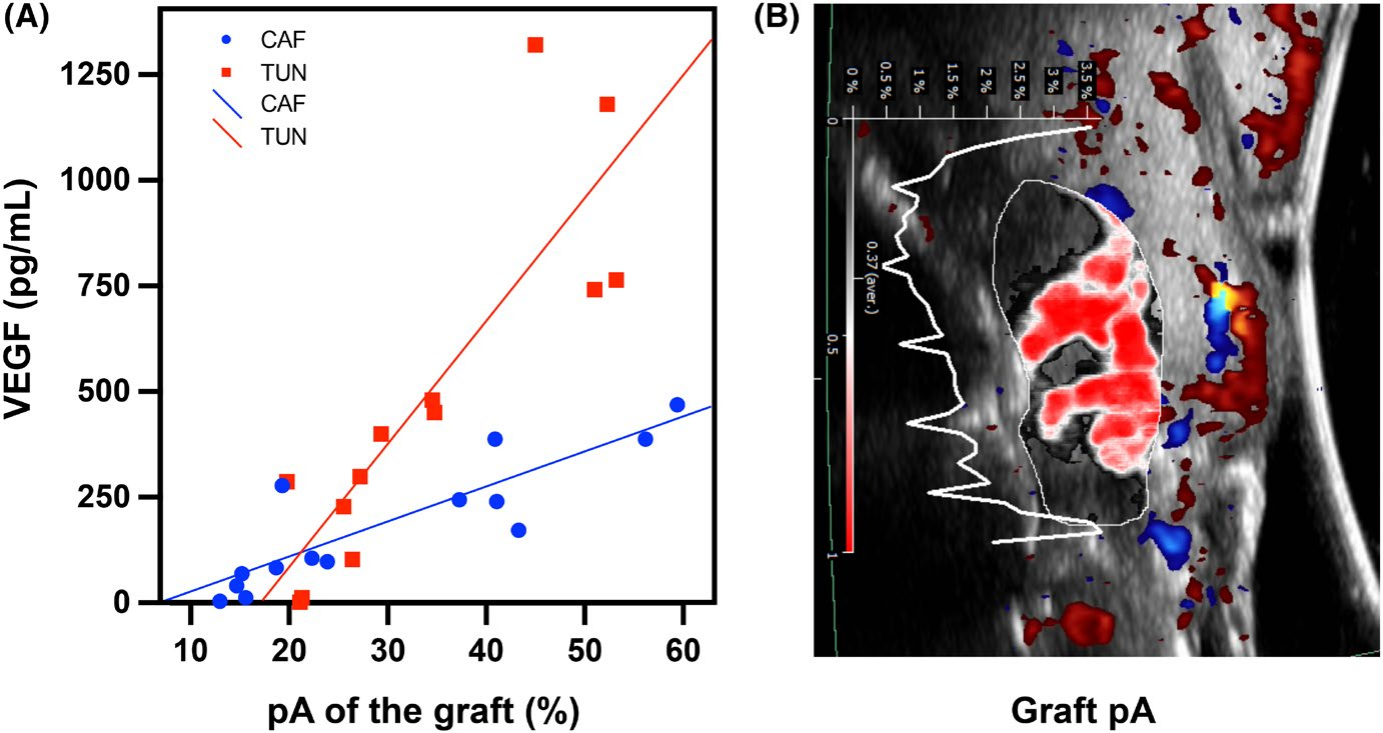
(A) Graph depicting the correlation between VEGF expression and tissue perfusion of the graft in terms of perfusion relief area (pA) at 1 week (*p* < .001 for both CAF and TUN). (B) Dynamic tissue perfusion assessment and pA quantification in an ultrasonographic scan obtained at the midfacial aspect of a dental implant 1 week after soft tissue augmentation.

A direct and significant correlation was observed between pRI of the graft and TIMP‐2 in both groups at 7 days (*p* < .001 in the CAF group, and *p* = .046 in the TUN group). VEGF levels and pA of the graft were found to be significantly correlated at the 7‐day time point (*p* < .001 for both CAF and TUN groups) (Figure 3).

## 4 DISCUSSION

Wound fluids have been extensively investigated in the medical field for identifying biomarkers associated with health vs. disease, disease progression, response to therapy, and healing outcomes.^27^, ^28^ Around dental implants, the analysis of proteins collected from the PICF has been mainly explored for identifying inflammatory biomarkers associated with peri‐implant disease^29^, ^30^, ^31^ and its resolution.^11^, ^32^

In the present study we have investigated the longitudinal expression of angiogenic biomarkers following peri‐implant soft tissue augmentation with CTG. Overall, we observed that the expression of all the investigated biomarkers was characterized by an initial peak at either day 3 or 7, followed by a decrease to baseline values. The longitudinal analysis revealed a statistically significant different expression of TIMP‐2 and VEGF over time between CAF‐ and TUN‐treated sites. It is possible that these findings reflect the differing healing processes of these two surgical procedures, with CAF requiring the incision of the interproximal soft tissue, in contrast to TUN that preserved of the integrity of the papillae. VEGF is a potent pro‐angiogenic factor that is also involved in several signaling pathways related to the vascular system and wound healing.^12^, ^15^ TIMP‐2 is known to play a key role in suppressing tissue proliferation in response to angiogenic factors.^33^, ^34^ The different slopes from curves depicting the return to baseline levels of VEGF and TIMP‐2 after the initial peaks, from both CAF‐ and TUN‐treated sites, further corroborates the different wound healing dynamic characterizing the two techniques. Other studies have shown a similar trend of VEGF, with an initial peak at the first or second week, followed by its decrease to baseline values after surgical procedures. Together, these findings support the role of VEGF in orchestrating early wound healing.^11^, ^12^, ^17^

Crevicular fluid biomarkers have been previously utilized in natural dentition to assess healing patterns following different soft tissue grafts and/or biologic agents.^3^, ^12^, ^14^, ^15^, ^16^, ^18^ Another interesting application of protein biomarkers is related to their ability to predict healing outcomes at later time points. Pellegrini et al. demonstrated that certain biomarkers could be used during early wound healing to identify better responders after periodontal regenerative procedures.^13^ In a clinical trial comparing the root coverage outcomes of CTG and collagen matrix, Skurska et al. observed correlations between changes in MMP‐1 and MMP‐8, and the final gain in keratinized tissue width and gingival thickness.^3^ In this study, we observed that VEGF levels at 7 days was significantly and directly associated with MT gain at 1 year. It is reasonable to assume that a greater vascularization of the graft at early time points, as indicated by the increase in VEGF expression, could have contributed to enhancing the outcomes of soft tissue augmentation. This may be achieved by a reduction in the amount of physiological contraction occurring within the soft tissue at later time points.

On the other hand, we noticed that elevated TIMP‐2 levels at the 3‐month follow‐up visit resulted in lower mean PSTD coverage and lower complete coverage of the dehiscence at the 1‐year mark. Although the initial increase of TIMP‐2 levels after surgery may be a physiological phenomenon, elevated values of this protein at later time points may indicate persistent inflammation and delayed healing at the treated sites, which may result in a contraction of the soft tissue with consequent apical shift of the mucosal margin.

Our findings also showed correlations between PROMs and the early expression of TIMP‐2. Patients with elevated levels of TIMP‐2 during the first week reported a shorter time to recovery. Serum levels of MMPs and TIMPs were found to play a role in pain perception after disc herniation surgeries.^35^ Preclinical studies demonstrated that MMPs are involved in postoperative and neuropathic pain.^36^ In particular, Fan et al. observed that the expression of MMP‐9/2 in the spinal cord increased significantly after plantar incision in mice, whereas the injection of a compound suppressing the activities of MMP‐9/2 relieved the induced allodynia.^37^ TIMPs are physiological inhibitors of MMPs that play a key role in angiogenesis, wound healing, inflammation, and tissue remodeling.^38^ The properties of TIMPs may explain the inverse correlation observed in our study between early expression of TIMP‐2 and time to recovery, such as elevated TIMP‐2 levels during the first week are associated with a shorter time to recovery, and possibly faster healing.

Our analysis also revealed that Doppler ultrasonographic outcomes correlates with the levels of angiogenic biomarkers (ANG, TIMP‐2, and VEGF) at 1 week post‐op. To the best of our knowledge, this is the first time that ultrasonographic tissue perfusion outcomes were demonstrated to be correlated with protein angiogenic biomarkers in our field. Doppler ultrasonography visualizes tissue perfusion using the acoustic signal phase‐shift effects, which is based on the relative frequency shifts of the received echoes as a result of the movement of red blood cells in the vessels.^10^ This technology has been largely applied in the medical field for discriminating normal blood flow from abnormal ones among other applications. Currently, it is considered to be a sensitive tool for the diagnosis of several pathological conditions.^39^, ^40^, ^41^ The combination of Doppler ultrasonography and angiogenic biomarkers assessment has been advocated for the evaluation and prediction of different clinical conditions.^42^, ^43^ Future studies should explore the value of the combined used of Doppler ultrasonography and protein biomarkers in the dental field for further assessing wound healing events, and for predicting pathological conditions and disease progression.

When interpreting our findings, clinicians should be aware that the original study was conducted with the main focus of comparing CAF vs. TUN for the coverage of PSTD, and therefore, it may be possible that the lack of statistically significant difference between the two groups for the levels of certain early wound healing biomarker could have been due to the limited statistical power. Future research on this topic should be designed with a sample size calculation based on biomarker levels and with a larger cohort that would possibly allow to further evaluate the influence of several patient and local factors on early wound healing outcomes.

## 5 CONCLUSION

The present study mapped the longitudinal local wound fluid‐associated expression patterns of PICF angiogenic biomarkers following CTG at implant sites. Within the limitations of the current research, a similar expression of ANG, FGF‐2, and PDGF‐BB was found between CAF and TUN groups over 3 months, whereas VEGF and TIMP‐2 levels exhibited significant differences at CAF‐ and TUN‐treated sites. TIMP‐2 and VEGF showed correlations with the 1‐year clinical and volumetric outcomes, as well as with post‐operative PROMs and Doppler ultrasonographic tissue perfusion‐related parameters. This study suggests that the combination of clinical measures, ultrasonography and real‐time biomarker assessment may aid in the better understanding of oral wound repair following soft tissue augmentation at dental implants.

## FUNDING INFORMATION

The study was supported by a grant from Delta Dental Foundation (AWD010089).

## CONFLICT OF INTEREST STATEMENT

The authors do not have any financial interests, either directly or indirectly, in the products or information enclosed in this manuscript.

## Supporting information


Appendix S1

## Data Availability

The data that support the findings of this study are available from the corresponding author upon reasonable request.
