# Supplementary material for: Characterization of oral biomarkers during early healing at augmented dental implant sites
Source: J Periodontal Res. 2024 Aug 1;60(3):206–14. doi: 10.1111/jre.13328 (PMC12024631; doi:10.1111/jre.13328)
Supplement: Supplementary file 1 — Appendix S1 [file JRE-60-206-s001.zip › Supplementary Table 1.docx]

**Supplementary Table 1.** Biomarker Expression at the CAF, TUN, and Lingual Sites Over 3 Months.

| **Group** | **Time**  **(days)** | **ANG**  **(mean ± SD) (pg/ml)** | **FGF-2**  **(mean ± SD)**  **(pg/ml)** | **PDGF-BB**  **(mean ± SD)**  **(pg/ml)** | **TIMP-2**  **(mean ± SD)**  **(pg/ml)** | **VEGF**  **(mean ± SD)**  **(pg/ml)** |
| --- | --- | --- | --- | --- | --- | --- |
| **CAF** | 0 | 243.11 ± 191.36 | 0.05 ± 0.10 | 0.53 ± 1.78 | 563.40 ± 248.73 | 51.40 ± 56.42 |
|  | 3 | 594.60 ± 201.05 | 3.53 ± 3.95 | 3.19 ± 3.90 | 918.52 ± 275.27 | 291.58 ± 292.62 |
|  | 7 | 465.84 ± 222.87 | 3.54 ± 3.14 | 1.57 ± 2.79 | 833.24 ± 224.61 | 185.67 ± 151.68 |
|  | 14 | 420.08 ± 219.80 | 1.86 ± 2.30 | 0.51± 1.76 | 812.56 ± 258.24 | 121.29 ± 114.51 |
|  | 30 | 237.61 ± 206.55 | 0.78 ± 1.15 | 0.33 ± 0.73 | 649.96 ± 342.30 | 96.49 ± 151.75 |
|  | 90 | 189.75 ± 92.39 | 0.33 ± 0.64 | 0.52 ± 1.79 | 387.05 ± 159.97 | 45.90 ± 49.88 |
| **TUN** | 0 | 221.94 ± 98.07 | 0.49 ± 1.08 | 0.15 ± 0.35 | 561.41 ± 213.27 | 72.94 ± 89.78 |
|  | 3 | 650.38 ± 195.62 | 5.48 ± 10.63 | 5.13 ± 7.40 | 883.47 ± 123.58 | 476.47 ± 403.48 |
|  | 7 | 489.38 ± 202.55 | 4.14 ± 12.83 | 2.45 ± 3.91 | 912.97 ± 238.26 | 482.75 ± 415.97 |
|  | 14 | 441.08 ± 190.54 | 2.24 ± 4.04 | 1.73 ± 3.41 | 847.68 ± 212.50 | 361.78 ± 355.33 |
|  | 30 | 276.68 ± 159.17 | 2.08 ± 6.69 | 0.69 ± 2.07 | 762.59 ± 316.53 | 167.09 ± 218.25 |
|  | 90 | 230.36 ± 125.15 | 0.16 ± 0.34 | 0.77 ± 1.38 | 696.33 ± 257.67 | 65.48 ± 62.16 |
| **Lingual sites** | 0 | 212.26 ± 162.07 | 0.26 ± 0.66 | 1.05 ± 2.79 | 467.84 ± 246.78 | 72.95 ± 78.88 |
|  | 3 | 433.40 ± 196.28 | 0.48 ± 1.21 | 2.30 ± 3.64 | 748.01 ± 296.30 | 276.74 ± 309.38 |
|  | 7 | 308.97 ± 92.65 | 0.88 ± 1.86 | 2.16 ± 4.25 | 642.45 ± 187.43 | 193.12 ± 246.19 |
|  | 14 | 261.78 ± 87.79 | 0.28 ± 0.77 | 1.47 ± 3.06 | 582.05 ± 176.97 | 126.25 ± 146.74 |
|  | 30 | 204.97 ± 113.99 | 0.51 ± 1.06 | 1.10 ± 2.04 | 456.27 ± 241.47 | 135.07 ± 159.99 |
|  | 90 | 194.86 ± 108.08 | 0.29 ± 0.83 | 0.82 ± 2.04 | 430.74 ± 183.56 | 65.79 ± 82.35 |

**Legend.** ANG: angiogenin. CAF: coronally advanced flap. FGF-2: fibroblast growth factor-2. PDGF-BB: platelet-derived growth factor-BB. TIMP-2: tissue inhibitor of metalloproteinases-2. TUN: tunnel technique. VEGF: vascular endothelial growth factor.
