# Supplementary material for: Characterization of oral biomarkers during early healing at augmented dental implant sites
Source: J Periodontal Res. 2024 Aug 1;60(3):206–14. doi: 10.1111/jre.13328 (PMC12024631; doi:10.1111/jre.13328)
Supplement: Supplementary file 1 — Appendix S1 [file JRE-60-206-s001.zip › Supplementary Table 2.docx]

**Supplementary Table 2**. Results of linear longitudinal regression assessing angiogenin (ANG) expression over time using generalized estimation equations model and control sites as reference category.

|  | **B** | **SE** | **95% Wald CI** | | **p-value** |
| --- | --- | --- | --- | --- | --- |
|  |  |  | **Lower** | **Upper** |  |
| **Intercept** | 301.1 | 19.0 | 263.9 | 338.4 | <0.001 |
| **TUN** | 141.6 | 25.5 | 91.5 | 191.7 | <0.001 |
| **CAF** | 119.2 | 36.8 | 47.0 | 191.4 | 0.001 |
| **Control** | 0 |  |  |  |  |
| **Time** | -1.38 | 0.36 | -2.10 | -0.66 | <0.001 |
| **TUN*Time** | -1.15 | 0.61 | -2.36 | 0.06 | 0.063 |
| **CAF*Time** | -1.39 | 0.54 | -2.45 | -0.33 | 0.010 |
| **Control*Time** | 0 |  |  |  |  |

**Legend**. B: estimated coefficient of the regression. CAF: coronally advanced flap. CI: confidence interval. SE: standard error. TUN: tunnel technique.
