# Supplementary material for: Characterization of oral biomarkers during early healing at augmented dental implant sites
Source: J Periodontal Res. 2024 Aug 1;60(3):206–14. doi: 10.1111/jre.13328 (PMC12024631; doi:10.1111/jre.13328)
Supplement: Supplementary file 1 — Appendix S1 [file JRE-60-206-s001.zip › Supplementary Table 3.docx]

**Supplementary Table 3**. Results of linear longitudinal regression assessing fibroblast growth factor-2 (FGF-2) expression over time using generalized estimation equations model and control sites as reference category.

|  | **B** | **SE** | **95% Wald CI** | | **p-value** |
| --- | --- | --- | --- | --- | --- |
|  |  |  | **Lower** | **Upper** |  |
| **Intercept** | 0.49 | 0.16 | 0.16 | 0.82 | 0.003 |
| **TUN** | 2.68 | 1.86 | -0.96 | 6.33 | 0.149 |
| **CAF** | 1.66 | 0.34 | 0.98 | 2.35 | <0.001 |
| **Control** | 0 |  |  |  |  |
| **Time** | 0 | 0 | 0 | 0 | 0.493 |
| **TUN*Time** | -0.03 | 0.01 | -0.06 | 0 | 0.092 |
| **CAF*Time** | -0.02 | 0 | -0.03 | 0.01 | 0.001 |
| **Control*Time** | 0 |  |  |  |  |

**Legend**. B: estimated coefficient of the regression. CAF: coronally advanced flap. CI: confidence interval. SE: standard error. TUN: tunnel technique.
