# Supplementary material for: Characterization of oral biomarkers during early healing at augmented dental implant sites
Source: J Periodontal Res. 2024 Aug 1;60(3):206–14. doi: 10.1111/jre.13328 (PMC12024631; doi:10.1111/jre.13328)
Supplement: Supplementary file 1 — Appendix S1 [file JRE-60-206-s001.zip › Supplementary Table 4.docx]

**Supplementary Table 4**. Results of linear longitudinal regression assessing platelet-derived growth factor-BB (PDGF-BB) expression over time using generalized estimation equations model and control sites as reference category.

|  | **B** | **SE** | **95% Wald CI** | | **p-value** |
| --- | --- | --- | --- | --- | --- |
|  |  |  | **Lower** | **Upper** |  |
| **Intercept** | 1.74 | 0.56 | 0.62 | 2.85 | 0.002 |
| **TUN** | 0.51 | 1.03 | -1.52 | 2.55 | 0.620 |
| **CAF** | -0.38 | 0.74 | -1.85 | 1.08 | 0.607 |
| **Control** | 0 |  |  |  |  |
| **Time** | -0.01 | 0 | -0.02 | 0.01 | 0.194 |
| **TUN*Time** | -0.01 | 0.01 | -0.03 | 0.01 | 0.536 |
| **CAF*Time** | 0 | 0.01 | -0.02 | 0.02 | 0.905 |
| **Control*Time** | 0 |  |  |  |  |

**Legend**. B: estimated coefficient of the regression. CAF: coronally advanced flap. CI: confidence interval. SE: standard error. TUN: tunnel technique.
