# Supplementary material for: Characterization of oral biomarkers during early healing at augmented dental implant sites
Source: J Periodontal Res. 2024 Aug 1;60(3):206–14. doi: 10.1111/jre.13328 (PMC12024631; doi:10.1111/jre.13328)
Supplement: Supplementary file 1 — Appendix S1 [file JRE-60-206-s001.zip › Supplementary Table 5.docx]

**Supplementary Table 5**. Results of linear longitudinal regression assessing tissue inhibitor of metalloproteinases-2 (TIMP-2) expression over time using generalized estimation equations model and control sites as reference category.

|  | **B** | **SE** | **95% Wald CI** | | **p-value** |
| --- | --- | --- | --- | --- | --- |
|  |  |  | **Lower** | **Upper** |  |
| **Intercept** | 604.9 | 28.6 | 548.8 | 660.9 | <0.001 |
| **TUN** | 190.7 | 40.5 | 111.3 | 270.2 | <0.001 |
| **CAF** | 190.1 | 49.5 | 93.1 | 287.2 | <0.001 |
| **Control** | 0 |  |  |  |  |
| **Time** | -2.16 | 0.54 | -3.22 | -1.10 | <0.001 |
| **TUN*Time** | 1.35 | 1.13 | -0.87 | 3.57 | 0.234 |
| **CAF*Time** | -2.16 | 0.74 | -3.61 | -0.70 | 0.004 |
| **Control*Time** | 0 |  |  |  |  |

**Legend**. B: estimated coefficient of the regression. CAF: coronally advanced flap. CI: confidence interval. SE: standard error. TUN: tunnel technique.
