# Supplementary material for: Characterization of oral biomarkers during early healing at augmented dental implant sites
Source: J Periodontal Res. 2024 Aug 1;60(3):206–14. doi: 10.1111/jre.13328 (PMC12024631; doi:10.1111/jre.13328)
Supplement: Supplementary file 1 — Appendix S1 [file JRE-60-206-s001.zip › Supplementary Table 6.docx]

**Supplementary Table 6**. Results of linear longitudinal regression assessing vascular endothelial growth factor (VEGF) expression over time using generalized estimation equations model and control sites as reference category.

|  | **B** | **SE** | **95% Wald CI** | | **p-value** |
| --- | --- | --- | --- | --- | --- |
|  |  |  | **Lower** | **Upper** |  |
| **Intercept** | 171.7 | 27.8 | 117.1 | 226.2 | <0.001 |
| **TUN** | 170.4 | 68.4 | 36.3 | 304.5 | 0.013 |
| **CAF** | -10.8 | 36.0 | -81.4 | 59.7 | 0.764 |
| **Control** | 0 |  |  |  |  |
| **Time** | -1.16 | 0.37 | -1.90 | -0.43 | 0.002 |
| **TUN*Time** | -1.90 | 0.83 | -3.54 | -0.26 | 0.022 |
| **CAF*Time** | -0.17 | 0.49 | -1.13 | 0.79 | 0.724 |
| **Control*Time** | 0 |  |  |  |  |

**Legend**. B: estimated coefficient of the regression. CAF: coronally advanced flap. CI: confidence interval. SE: standard error. TUN: tunnel technique.
